# Cognitive Aspects of Generalised Anxiety Disorder In Adolescents: Exploring Intolerance of Uncertainty, Cognitive Avoidance, and Positive Beliefs About Worry

**DOI:** 10.1007/s10578-025-01809-3

**Published:** 2025-02-25

**Authors:** Lottie Shipp, Eleanor Leigh, Amy Laverton, Ray Percy, Polly Waite

**Affiliations:** a Department of Experimental Psychology, University of Oxford, Woodstock Rd, OX2 6GG; b School of Psychology and Clinical Language Sciences, Harry Pitt Building, University of Reading, Earley Gate, Reading, RG6 6ES

**Keywords:** Generalized Anxiety Disorder, intolerance of uncertainty, adolescence, mental health, metacognitive beliefs

## Abstract

Intolerance of uncertainty, cognitive avoidance, and positive beliefs about worry have been well researched in adults with Generalised Anxiety Disorder (GAD), but few studies have explored these aspects in adolescent clinical samples. To address this gap, we compared the three cognitive variables amongst 12-18 year-olds with diagnoses of GAD (*n* = 46), those with other anxiety disorders (*n* = 18), and a group with low self-reported anxiety (*n* = 38). Adolescents with GAD reported significantly higher intolerance of uncertainty and positive beliefs about worry relative to their peers with other anxiety diagnoses, whilst levels of cognitive avoidance did not differ significantly. Intolerance of uncertainty and cognitive avoidance, but not positive beliefs about worry, were significantly higher amongst young people with GAD compared to the non-anxious community group. This novel study provides preliminary evidence for the relevance and specificity of intolerance of uncertainty to the maintenance of GAD in adolescence.

## 1 Introduction

Anxiety disorders are common in childhood and adolescence [1; 2] and are associated with significant distress and functional impairment [3]. Generalised Anxiety Disorder (GAD) is characterised by uncontrollable worry and somatic symptoms (e.g., muscle tension [4]). It affects 1-3% of 11-19 year-olds [5], commonly co-occurs with other anxiety disorders [6], and is one of the most frequent anxiety diagnoses in treatment-seeking adolescent populations [7]. There is an increase in the prevalence [5] and clinician-rated symptom severity [7] of GAD from childhood into adolescence, perhaps reflecting the growth of meta-cognitive skills [8], and the heightened social-evaluative and academic concerns [9, 10] associated with this developmental stage. Yet despite its prevalence and negative effects, there is a lack of research into GAD relative to other anxiety disorders [11].

Across the lifespan, the hallmark feature of GAD is pervasive worry. This is defined as “a chain of thoughts and images, negatively affect-laden and relatively uncontrollable” (p.10) that is typically future-focused and reflective of an attempt at mental problem-solving [12]. It often presents as “what if…?” cognitions in which potential impending threats and uncertainties are identified [13]. Whilst worry exists on a continuum from normal to pathological [14], with the same content in individuals with GAD and those without [15], a GAD diagnosis is associated with significant emotional distress and impairment relating to worry [16]. Additionally, it has been suggested that clinical and non-clinical worriers are differentiated by the factors that underly their problematic cognitions, such as safety-seeking behaviours and threat beliefs [17]. Thus, it is vital to explore not *what* young people with GAD worry about, but *how* and *why* they worry. By furthering our understanding of the aspects that maintain this disorder, we can inform the development of effective, targeted psychological interventions [18]. This is necessary as most youth with GAD currently receive a disorder-general form of cognitive-behavioural therapy (CBT) which achieves remission rates of just 50% [19]. This leaves a concerning number of young people who continue to meet diagnostic criteria after treatment, and highlights the need for further research in this field.

A number of theoretical models of GAD in adults have been proposed to explain its aetiology and maintenance, and several have informed effective treatment approaches (see [20] for a review). For example, the Intolerance of Uncertainty Model [15; 21] suggests that individuals with GAD hold a set of negative beliefs about uncertainty and its consequences [22]. Felt uncertainty is appraised as a sign of potential danger; it drives positive beliefs about worry, sets in motion a spiral of worrisome thinking, and feeds a negative problem orientation. Together, these cognitive, behavioural, and emotional processes exacerbate worry and motivate use of avoidance strategies (e.g., thought suppression). Treatments based on this model involve worry awareness training and re-evaluation of faulty beliefs about worry [22], and have been shown to result in large decreases in GAD severity, worry, and intolerance of uncertainty in adults [23]. Similarly, a separate extant model proposed by Wells [24] states that in GAD, positive beliefs about the usefulness of worry motivate its use as a coping strategy whilst concurrent negative beliefs about its danger (e.g., “I will lose my mind with worry”) cause anxiety to escalate. A vicious cycle is set in motion, as the cognitive and physiological symptoms associated with the worry reinforce problematic metacognitive beliefs and behaviours.

Furthermore, Borkovec et al. [25] conceptualise worry as a cognitive avoidance strategy which, due to its verbal-linguistic nature, can inhibit negative mental images and their associated aversive somatic and emotional experiences. Via this process, worry hinders the emotional processing of fear-based information that is essential for extinction and habituation of the anxiety response [26], and positive beliefs about worry are reinforced by the avoidance of unwanted sensations [27].

Alternatively, Gustavsson et al.’s [28; 17] recent model of GAD in adults suggests that worry should be viewed as a safety-seeking behaviour which occurs in the context of negative core beliefs about oneself (e.g., “I’m a failure”). Its function is to reach a state of absolute certainty by foreseeing and taking steps to prevent all potential negative outcomes, which if they occurred, would confirm negative beliefs. GAD is maintained as the desired level of absolute certainty can never be realistically achieved, and failure of the feared outcome to take place reinforces positive beliefs about worry.

These models provide insight into the mechanisms underlying GAD in adults; however, little work has explored whether the same cognitive aspects are associated with the disorder in adolescence. This cannot be assumed, as adolescence is a unique developmental stage associated with distinct biological, social, and psychological factors [29]. Some preliminary evidence suggests that the aspects identified in adult cognitive models of GAD are also relevant in younger populations – for instance, relationships have also been found between cognitive avoidance and worry or anxiety in non-clinical adolescents [30; 31]. However, other findings are mixed as demonstrated by a combination of significant [32; 31] and non-significant [32] results regarding positive beliefs about worry.

Furthermore, studies are often limited by their use of community populations (e.g., [34; 30; 32; 35]), or inclusion of clinical samples with broad age ranges spanning childhood and adolescence (e.g., [36; 37]). Of the clinical research, worry is frequently explored in the context of anxiety disorders in general, and therefore conclusions are not GAD-specific (e.g., [38]).

Additionally, questions remain regarding the specificity of these cognitive aspects to GAD (e.g., [39]) – for example, intolerance of uncertainty explains additional variance in social anxiety symptoms after accounting for neuroticism, fear of negative evaluation, and anxiety sensitivity in adults [40] and individuals with specific phobias report greater efforts to engage with thought suppression than non-phobic controls [41]. It is vital to explore the question of specificity in order to understand the maintenance of GAD, facilitate accurate diagnoses [42], and inform more effective treatments approaches [43].

The present study examined levels of intolerance of uncertainty, cognitive avoidance, and positive beliefs about worry in adolescents. It addresses the shortcomings of previous studies with the inclusion of treatment-seeking young people who had GAD as their primary or secondary diagnosis (i.e., GAD was present in their diagnostic profile but was not the main presenting problem). The study also included adolescents with other anxiety disorders (without GAD in their diagnostic profiles). This ‘other anxiety disorder’ control group allowed us to assess whether the three cognitive aspects of interest are unique to GAD, or if they are general elements related to anxiety more broadly (with the former consistent with, although not sufficient evidence for, a causal role in GAD). To complete this analysis, scores were also compared to a community group who self-reported low levels of anxiety.

Our hypotheses were as follows:

1) Adolescents with primary or secondary GAD will show significantly higher levels of a) intolerance of uncertainty, b) cognitive avoidance, and c) positive beliefs about worry than adolescents with other anxiety disorders.
2) Adolescents with primary or secondary GAD will show significantly higher levels of a) intolerance of uncertainty, b) cognitive avoidance, and c) positive beliefs about worry than adolescents with low self-reported anxiety recruited in the community.
3) Intolerance of uncertainty, cognitive avoidance, and positive beliefs about worry will predict significant variance in GAD symptoms within the group of adolescents with primary or secondary GAD.

## 2 Methods

### 2.1 Study Design

This cross-sectional study included three groups of young people: those with a primary or secondary diagnosis of GAD (subsequently referred to as the ‘GAD group’), those with other anxiety disorders without GAD in their diagnostic profile (‘anxiety control group’), and non-anxious community participants (‘community group’). All participants completed the same battery of self-report measures.

### 2.2 Participants

Data from 102 adolescents aged 12-18 years (mean age = 15.12 years, SD = 1.82) were analysed in this study (see Table 1 for demographic and clinical characteristics).

Young people in the two clinical groups, recruited through local child and adolescent mental health services (CAMHS), were eligible if they met criteria for a primary anxiety disorder according to the Anxiety Disorder Interview Scale - Child/Parent version [44]. They were ineligible if they had an established autistic spectrum disorder, suicidal intent, recurrent and deliberate self-harm, or if they were identified as currently ‘at risk’ due to child protection concerns. To be eligible for the community group, adolescents were required to score below the clinical cut-off (T-score of 65) on the total anxiety subscale of the Revised Children Anxiety and Depression Scale (RCADS; [45]). Given resource constraints, it was not possible to deliver diagnostic interviews to this group. Across all three groups, young people were ineligible if they had learning disabilities that would pose a barrier to completing self-report measures.

### 2.3 Procedure

The study followed ethical standards, and was granted approval by the University of Reading’s Research Ethics Committee (reference: 15/27) and the NRES Committee South Central – Berkshire B (reference: 15/SC/0081). The analysis plan was pre-registered on the Open Science Framework (https://doi.org/10.17605/OSF.IO/UGN7F)^1^.

The clinical groups were recruited through a local NHS-commissioned CAMH service between December 2015 and February 2020. To be eligible for a referral, the young person had to be experiencing symptoms of anxiety and/or depression that caused significant distress or impairment (i.e., likely to meet diagnostic criteria). If the referral indicated a high likelihood of eligibility for the study, adolescents and their parents/carers were provided with information sheets, and a diagnostic assessment took place. This was conducted by assessors (clinicians or placement students) who were trained to high levels of reliability and received regular supervision from a senior clinician with extensive experience.

If they met criteria for a primary anxiety disorder, the study was discussed with adolescents and their parents at their next appointment. Young people aged 12-15 years provided written assent and their parents/carers provided written consent, whereas those aged 16-18 years provided written consent. If consent/assent to participate was provided, self-report measures were completed on paper or online through a secure system.

The community group was recruited through local schools between March and August 2016. Those who registered their interest were sent information sheets and contacted by a member of the research team to address any questions. If consent/assent to participate was provided, adolescents were given the questionnaires to complete.

Participants were reimbursed for their time with a £10 gift voucher, and were provided with information about sources of support in case of any concerns arising after the study.

### 2.4 Measures

#### Demographic Information

Participants provided basic demographic information: age, gender, ethnicity, and parent/guardian education and occupation (used as a proxy measure for socioeconomic status (SES)). Whilst we aimed to collect ethnicity data for the community group from schools, this was not possible and therefore this information is only available for the clinical group.

#### Diagnostic Assessments (clinical groups only)

Adolescents’ current anxiety disorder diagnoses were assessed using the Anxiety Disorder Interview Schedule (ADIS-IV-C/P [44]; a gold-standard semi-structured interview adapted for DSM-5 with good to excellent psychometric properties in child and adolescent populations [46]. Clinician Severity Ratings (CSRs) are assigned on a 9-point scale from 0 (no impairment) to 8 (severe impairment). A CSR of 4 or more (based on self- or parent-reported symptoms) indicates a diagnosis. In the case of multiple diagnoses, the disorder with the highest CSR is classed as the primary problem. In this study, co-occurring disorders (e.g., somatoform and substance abuse disorders) were also assessed using the ADIS-C/P. The Kiddie Schedule for Affective Disorders and Schizophrenia Present and Lifetime (K-SAD-S-PL; [47]) was used to assess whether adolescents met DSM-5 criteria for co-occurring Major Depressive Disorder (MDD) and Mania, as this semi-structured assessment tool is considered the gold-standard for evaluating mood disorders in young people (e.g., [48]). Diagnoses are dependent on a synthesis of both self- and parent-reported data. Although not typically used in the K-SADS-S-PL, CSRs were included to ensure consistency across anxiety and depression diagnoses. The K-SAD-S-PL has been shown to have good to excellent test-retest reliability in child and adolescent populations [47].

Overall inter-rater reliability for diagnostic assessments was reviewed for a subsample of 85 assessments from a wider study, including 97% of the clinical participants whose data were included in the current analyses. Inter-rater reliability was good for 94% of diagnoses (comprising those that were more common), and poor for the remaining 6% (comprising less frequent diagnoses – e.g., obsessive-compulsive disorder, illness anxiety disorder, and post-traumatic stress disorder). Therefore, it was deemed to be good overall.

#### Self-Report Measures (clinical and community groups)

##### Intolerance of Uncertainty Scale for Children (IUSC; [49])

The IUSC is a 27-item self-report measure of emotional, cognitive, and behavioural responses to situations of uncertainty. It is a version of the adult Intolerance of Uncertainty Scale [50], adapted to be developmentally appropriate for younger populations. Respondents rate the degree to which statements (e.g., “Not knowing what will happen in the future makes me uneasy, anxious, or stressed”) describe themselves on a 5-point scale from “Not at all” to “Very much”. Scores are summed to give a total in the range of 27 - 135, with higher scores indicative of greater intolerance of uncertainty. The IUSC has been found to have excellent internal consistency in community and clinical samples of children and adolescents (Cronbach’s α = 0.91 - 0.94; [49]). This was replicated in the current sample, with Cronbach’s α = 0.97 and McDonald’s ω = 0.98.

##### Cognitive Avoidance Questionnaire (CAQ [51; 52]).

The CAQ is a 25-item self-report measure, which addresses 5 cognitive avoidance strategies: Thought Suppression, Thought Substitution, Distraction, Avoidance of Threatening Stimuli, and Translation of Images Into Thoughts. Items (e.g., “I have thoughts that I try to avoid”) are rated on a 5-point scale from 1 (“Not at all typical”) to 5 (“Completely typical”). Items are summed to give a total in the range of 25 - 125, with higher scores indicating a greater use of cognitive avoidance strategies. The CAQ has been shown to have excellent internal consistency in adolescent community populations [51]. In the current sample, Cronbach’s α = 0.95 and McDonald’s ω = 0.96.

##### Why Worry-II (WW2; [53])

The WW2 is a version of the original Why Worry? scale (WW; [50]), including 25 self-report items addressing positive beliefs about worrying. In this study, we adapted items to ensure suitability of language for young people – for example, item 24 was amended from “By not worrying, one can attract misfortune” to “By not worrying, things can go wrong”. Items are rated on a 5-point scale, and load onto 5 subscales: (1) worry aids problem solving; (2) worry increases motivation; (3) worry protects against difficult emotions; (4) worry itself prevents the occurrence of negative outcomes; and (5) worry reflects a positive personality trait. Responses are summed to give totals in the range 25-125, with higher scores indicating greater perceived benefit of worrying. The WW2 has demonstrated excellent internal consistency in non-clinical adolescent populations (Cronbach’s α = 0.90; [32]). Due to an administrative error, item 2 of the WW2 was incorrectly worded on the questionnaires in the present study, and therefore the total reported here is the sum of the 24 remaining items (with sum scores ranging from 24-120). This did not negatively affect the internal consistency of the scale, as demonstrated by Cronbach’s α = 0.94 and McDonald’s ω = 0.95.

##### Revised Children Anxiety and Depression Scale (RCADS; [54])

The 47-item RCADS includes 6 subscales, assessing separation anxiety disorder, social anxiety disorder, generalised anxiety disorder, panic disorder, obsessive compulsive disorder, and major depressive disorder. The 5 anxiety subscales can be combined to generate a total anxiety score (ranging from 0-141). Items are rated from 0 (“Never”) to 3 (“Always”). In a clinical sample of 7-17 year-olds, the RCADs had good internal consistency (Cronbach’s α = 0.78 - 0.88), a cut-off score of 7 on the GAD subscale resulted in a sensitivity of 0.69 and a specificity of 0.72, and its factorial validity was supported [46]. In the current sample, Cronbach’s α ranged from 0.83 – 0.96 and McDonald’s ω ranged from 0.91 – 0.97 for the total anxiety subscale (used to screen community participants), and GAD and MDD subscales (used as measures of GAD and MDD symptoms, respectively).

### 2.5 Data Analysis

#### 2.5.1 Power Analyses

Power analyses were conducted using G*Power v.3 [55] for 95% power (alpha = 0.05 two-sided) based on Read et al.’s [37] data. This study compared 7-17 year-olds with and without GAD according to intolerance of uncertainty, and reported an effect size of *r* = 0.73. It was calculated that 42 participants in each group would be required; as the final sample was smaller than this, our study was underpowered. This was particularly notable for the anxiety control group (*n* = 18), which had less than half the target number of participants.

#### 2.5.2 Analysis Plan

**Fig 1:**
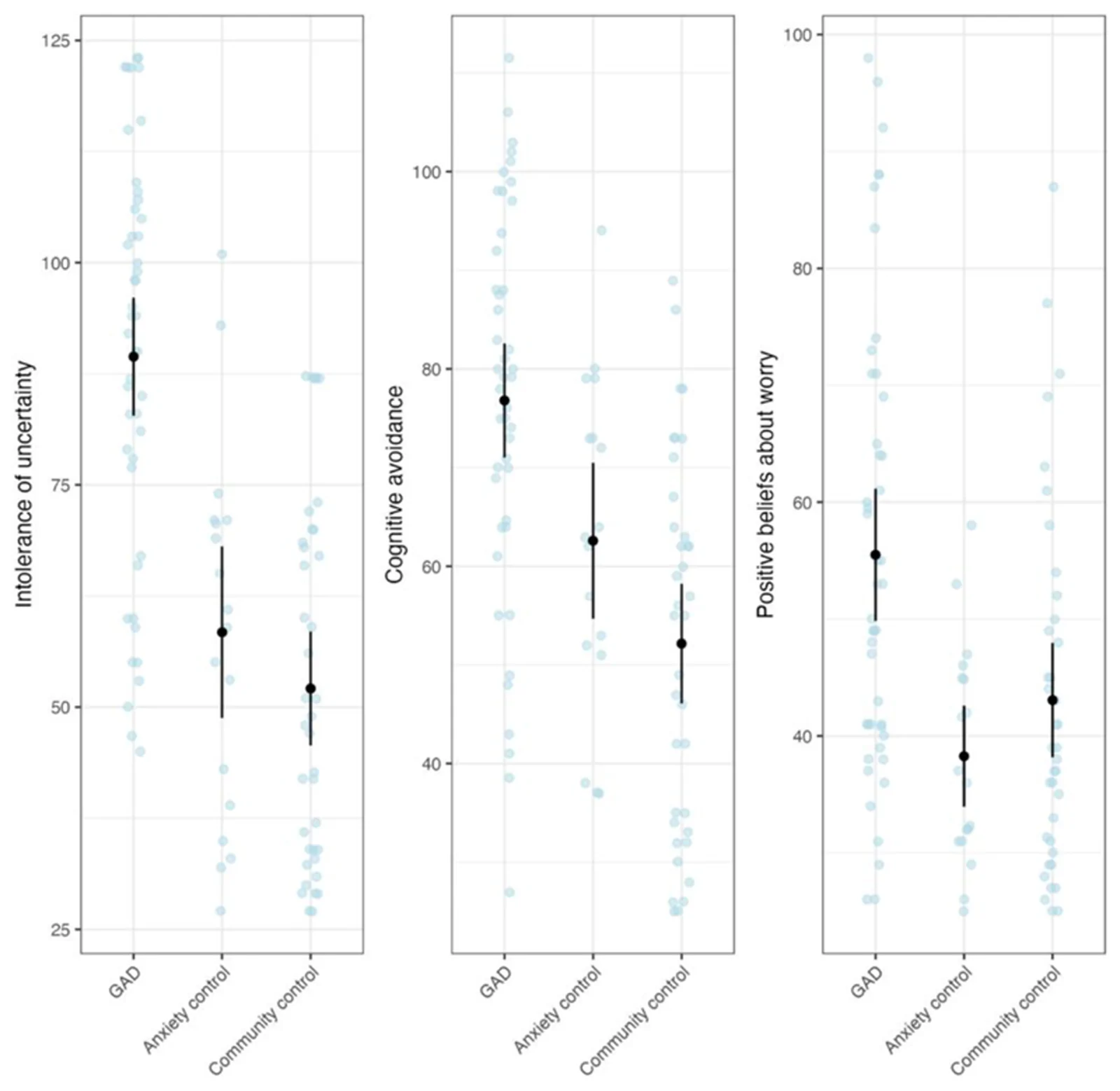
Cognitive aspect measures by group. Blue circles represent individual data points. Black circles represent mean values, and black vertical lines represent 95% confidence intervals

Data were analysed with SPSS v.29 [56] and RStudio v.2023.09.0+463 [57]. Figure 1 was created with the ggplot2 [58] and patchwork [59] packages for RStudio. McDonald’s ω was calculated with the psych package [60].

Chi-square tests were conducted to compare the proportion of females across the three groups. Independent samples t-tests checked for group differences in age and CSR.

Hypotheses 1 and 2 were assessed with linear regression analyses, with IUSC, CAQ, and WW2 scores as the dependent variables (included in separate analyses), and gender, age, and depression symptoms included as covariates. Assumptions (including homoscedasticity, linearity, homogeneity, and the absence of outliers and multicollinearity) were assessed with the check_model functions from the performance package [61]. A stepwise multiple linear regression model, conducted with the lm function in R Studio, was used to assess Hypothesis 3. Bootstrapping was applied, and the boot.pval package [62] was used to generate co-efficient estimates, confidence intervals, and *p*-values.

Exploratory analyses were conducted in order to assess Hypotheses 1 and 2 for only those with primary GAD (*n* = 18) relative to the anxiety and community groups.

#### 2.5.3 Missing Data

Questionnaires with more than 10% of items missing were classed as incomplete, and only complete cases were included in the final sample. When less than 10% of items were missing, individual-level item mean substitution was performed [63; 64]. Visual assessment indicated that missing data was not associated with key characteristics (e.g., gender, age, and SES; see Supplementary Materials, Table S1).

## 3 Results

### 3.1 Demographics

The GAD group had a significantly higher proportion of girls/young women (95.7%) than the anxiety control (44.4%; χ^2^ (1) = 22.27, *p* < 0.001) and community groups (44.7%; χ^2^ (1) = 27.13, *p* < 0.001). There were no significant age differences between the GAD and anxiety control group (*t*(62) = -0.31, *p* = 0.755), or between the GAD and community group (*t*(59.72) = 6.3, *p* = 0.529). The CSR of primary diagnosis did not differ significantly between the two clinical groups (*t*(61) = -1.04, *p* = 0.205).

### 3.2 Symptom and cognitive process measures

Means, standard deviations, and 95% confidence intervals for symptom and/or cognitive measures are reported in Table 2 and Figure 1.

### 3.3 Hypothesis 1

After controlling for gender, age, and depression, there were significant differences between the GAD and anxiety control groups in relation to intolerance of uncertainty and positive beliefs about worry (*p* < 0.001 and *p* = 0.003 respectively), such that the GAD group scored higher on both measures. The difference between the groups regarding their level of cognitive avoidance fell short of significance (*p* = 0.057). Adjusted *R*^*2*^ ranged from 0.172-0.435, indicating that the model explained the greatest amount of variance in intolerance of uncertainty (43.5%), followed by positive beliefs about worry (32.8%), and cognitive avoidance (17.2%). Greater levels of depression significantly predicted higher scores on all three cognitive variables (see Table 3).

### 3.4 Hypothesis 2

After controlling for gender, age, and depression, there were significant differences in intolerance of uncertainty (*p* = 0.005) and cognitive avoidance (*p* = 0.033) between the GAD and community groups, such that the GAD group scored higher on both measures.

There were no significant differences with respect to positive beliefs about worry (*p* = 0.929) and in all three analyses, depression was predictive of higher cognitive aspect scores (see Table 4). The pattern of results remained unchanged after applying the Benjamini-Hochberg correction with a 10% false discovery rate to control for Type 1 errors due to multiple comparisons (see Supplementary Materials, Table S2). Adjusted *R*^*2*^ revealed that the model accounted for the greatest amount of variance in intolerance of uncertainty (60.8%), followed by cognitive avoidance (43.9%), and positive beliefs about worry (32.9%).

### 3.5 Hypothesis 3

Given violations of the assumptions of linear models, and the lack of success of log and square root transforms, bootstrapping was used as this technique is more robust than parametric approaches [65].

A bootstrapped multiple linear regression using a forward stepwise method was conducted, with age, gender, depression, IUSC, CAQ, and WW2 scores as predictors of GAD symptoms within the GAD group. The resulting model dropped age and gender, and none of the remaining variables predicted significant variance in GAD scores (see Table 5).

### 3.6 Exploratory analyses

Exploratory analyses were conducted in which only participants with primary GAD were included in the GAD group (see Supplementary Materials, Tables S3 and S4). There were no significant effects of group on any of the cognitive variables after applying the Benjamini-Hochberg method (see Table S2).

## 4 Discussion

This study explored whether three cognitive aspects that have been identified in adult models of GAD (intolerance of uncertainty, cognitive avoidance, and positive beliefs about worry) were elevated in adolescents with primary or secondary GAD, relative to anxiety control and community groups. Our hypotheses were partially supported. The GAD group was significantly more intolerant of uncertainty than both the anxiety control and community groups. Whilst cognitive avoidance was significantly higher in the GAD group compared to the community group, the comparison with the anxiety control group fell short of significance. In contrast, positive beliefs about worry were significantly higher in the GAD group compared to the anxiety control group, but not the community group. The three cognitive variables did not predict significant variance in GAD symptoms within the GAD group. Additionally, exploratory analyses revealed no significant group differences on cognitive variables when only those with a primary diagnosis of GAD were included in the GAD group.

Elevated intolerance of uncertainty in adolescents with GAD aligns with existing research in community populations [35; 30; 32; 34] and further supports a role for this construct in clinical samples. The significantly higher intolerance of uncertainty in the GAD group compared to the clinical control group can be interpreted as evidence for the specificity of this cognitive process to GAD. Mean intolerance of uncertainty scores were relatively similar for the anxiety control and community groups (*M* = 58.42 and *M* = 52.09, respectively) compared to the GAD group (*M* = 89.43), further supporting a unique relationship between intolerance of uncertainty and GAD. This supports previous research in adults showing that high intolerance of uncertainty distinguishes GAD from panic disorder with agoraphobia [66] and other anxiety disorders more broadly [67]. Moving forwards, studies may benefit from turning attention to the factors driving intolerance of uncertainty. For instance, one possible direction for research is the threat beliefs associated with GAD, which are suggested to centre around an intense fear of failure and a belief in one’s inability to cope [68; 69]. Such negative threat beliefs could underly an intolerance of uncertainty and associated positive views of worry [28] - for example, the notion that “Worry helps me cope” could be underpinned by a core negative belief about oneself (e.g., “I am incompetent” [70]). The role of such threat beliefs has received little research attention in GAD [17], but they are likely to be particularly relevant for adolescents who may be more vulnerable to fears of failure and negative evaluation, insecurity, and critical internal dialogues [71; 72].

Aligning with previous findings in adult populations [67], adolescents with GAD reported significantly greater use of cognitive avoidance than those in the community group. However, the difference between the GAD and anxiety control groups failed to achieve significance by a marginal amount. Whilst possible that this result is reflective of the ubiquity of avoidance behaviours across anxiety disorders, it may also be accounted for by our lack of statistical power. It might be that although avoidance is common across anxiety disorders, *cognitive* avoidance is more characteristic of GAD whereas other disorders are associated with greater *behavioural* avoidance. For example, whilst an individual with panic disorder who has previously had a panic attack on public transport may be able to avoid this situation, someone with GAD who worries about a great number of everyday events is likely to be less able to avoid anxiety-provoking circumstances completely. Instead, they may use cognitive avoidance strategies to control their anxiety within the situation. This is an area for future research, with larger samples and thus greater statistical power. The different patterns of significance between the intolerance of uncertainty and cognitive avoidance constructs imply that the former is more specific to GAD, whereas the latter is a process that is common across the anxiety disorders. Furthermore, both intolerance of uncertainty and cognitive avoidance are elevated in adolescent girls relative to boys [30]; given the gender imbalance in the GAD group, this may have contributed to stronger effects. This imbalance may be the result of boys experiencing greater barriers to accessing clinical services [81]. As such, future work must aim to overcome these obstacles. By increasing representation in clinical services (and by extension, research) we would be better placed to explore the effects of gender on anxiety.

Results showed significantly higher levels of positive beliefs about worry in the GAD group compared to the anxiety control group. This aligns with multiple cognitive models of GAD, which share the view that worry is reinforced by positive beliefs about its necessity [12; 15; 28; 24].

Interestingly, we found no significant difference between the GAD and community groups with respect to positive beliefs about worry, in contrast to previous research in adults (e.g., [67]). This finding may plausibly be the result of similar responses being given in different contexts across groups. For instance, “By worrying, I can find a better way to do things” may be true of someone who experiences time-limited and controlled worry. Yet equally, this belief could be held by an adolescent with GAD who worries excessively about finding the perfect solution to every perceived problem, and is unable to terminate the worry process until this unrealistic goal has been achieved. It could be inferred that where positive beliefs relate to pathological worry, this may contribute to the maintenance of GAD in young people. However, the small difference in mean scores between the anxiety control and community control group implies a relatively small effect of anxiety or community control group membership on this measure. This aligns with Wells’ metacognitive model [24], which proposes that positive beliefs about worry are not unique to GAD. Rather, negative beliefs (e.g., that worry is uncontrollable and dangerous) differentiate GAD from social phobia and panic disorder [73]. Consequently, future studies may wish to explore whether negative beliefs about worry show greater specificity to GAD.

In all three analyses, depression was associated with significantly higher scores on cognitive variables. This is likely due to overlap between the mechanisms associated with worrying in the context of GAD, and those linked with depressive rumination. This can be understood by their shared characteristics (e.g., uncontrollability and negative content), and is reflected in the fact that both fall under the umbrella of ‘repetitive negative thinking’ (RNT; [74]). However, the finding that the presence of GAD predicted higher scores on cognitive aspects beyond the effects of depression supports their conceptualisation as separate disorders that may share cognitive patterns, but are nonetheless distinct [75]. This result has implications for debates regarding the nosology of GAD and depression [76], and provides avenues for further research into factors that distinguish GAD from other internalising disorders. Future studies may also wish to explore how GAD presents in isolation relative to when it co-occurs with depression – for example, with use of a two-by-two factorial design. Whilst extant research has assessed the differential impairments associated with pure and co-occurring GAD (e.g., [80]), whether cognitive processes differ between the two diagnostic groups remains relatively unexplored despite having clear clinical relevance.

Contrary to hypotheses, cognitive variables did not predict significant variance in GAD symptoms within the GAD group after controlling for gender, age, and depression. This may indicate that whilst these factors are relevant to GAD, they are not significant predictors of its severity. However, it is also possible that this result is due to a lack of statistical power or a failure of the outcome measure (RCADS-GAD subscale) to capture generalised anxiety symptoms. Exploratory post-hoc analysis revealed that of the participants with a diagnosis of primary or secondary GAD (determined by a gold-standard clinical interview), only 8 (17.4%) scored in the clinical range on this subscale. This may be because 5 of its 6 questions relate to pathological worry which, although a key feature of GAD, is not its only characteristic [16]. Thus, future research may benefit from using a different measure that reflects additional aspects of GAD (e.g., somatic symptoms).

Exploratory analyses comparing only participants with primary GAD to the control groups revealed no significant differences on any of the three cognitive measures – a result that may plausibly be accounted for by a lack of statistical power.

The strengths of this study include its clinical population, use of diagnostic interviews for the clinical groups, and ability to assess the extent to which these cognitive aspects are unique to GAD relative to other anxiety disorders (i.e., to establish broad specificity [42]).

Yet despite these merits, it is important to interpret findings in light of their limitations. Notably, the study was underpowered; while we were able to successfully recruit adolescents with GAD and adolescents from the community, it was challenging to recruit young people from clinical services with anxiety disorders where they did not have GAD in their diagnostic profiles, reflecting the high prevalence and pervasiveness of GAD in this age group. It is possible that has this group been larger, this would have changed the results, for example, the difference between this group and the GAD group for cognitive avoidance may have reached significance. This highlights the need for further research and to establish the relevance of our findings to a large proportion of young people. GAD is one of the most common anxiety diagnoses [5; 7], yet accounts for only 8% of anxiety disorder publications [79]. As such, moving forwards GAD should receive more research attention in line with its high prevalence and burden.

Furthermore, the strong gender bias in the GAD group was disproportionate to what would be predicted on the basis of prevalence rates [5]. Girls report higher levels of worry than boys [30], and thus it is possible that the greater proportion of girls in the GAD group compared to both control groups could partially account for our pattern of results. As such, future studies with larger and more balanced samples should be conducted to explore potential gender differences. Furthermore, due to resource constraints, we were unable to deliver diagnostic assessments to the community control group, instead screening on the basis of self-reported anxiety symptoms. The total anxiety score from the RCADS was used for this purpose; however, it does not assess all the anxiety disorders included in the DSM-5 [4]. As such, this assessment process was less rigorous than a clinical interview, leaving a possible presence of psychological disorders. Whilst this introduced a degree of uncertainty regarding the clinical characteristics of this subgroup, it also represents a more conservative comparison and thus may even lend stronger support to our significant results. We were also limited in the number of variables we could examine and therefore future research should consider additional factors, such as negative problem orientation [15].

Finally, our cross-sectional design means that we cannot determine whether, for example, intolerance of uncertainty has causal or consequential specificity to GAD [42]. Therefore, there is a need to replicate and build upon our findings in well-powered longitudinal studies with more diverse populations. It may also be beneficial to explore if and how the cognitive profile of GAD differs across the lifespan. This could involve directly comparing treatment-seeking samples of adolescents, children, and adults to provide a nuanced insight into any developmental differences and their effects on clinical characteristics.

Our novel findings indicate the relevance of intolerance of uncertainty and its specificity to GAD in adolescence, and may have clinical implications. Treatments for GAD that specifically aim to increase tolerance of uncertainty draw on Dugas et al.’s cognitive model [15], and include components such as worry awareness training and exposure to unsure situations. Preliminary studies of these interventions for adolescents have reported variable results ranging from reliable clinical improvement in 3 out of 7 participants [77] to 7 out of 12 participants [38]. Therefore, further work may include optimising treatments before comparing them in larger scale, more controlled trials. Overall, the picture of treatment for GAD in adolescents highlights the need for a better understanding of the mechanisms that maintain this anxiety disorder, and the refinement of targeted interventions that successfully address these processes.

## Supplementary Material

Supplementary Materials

## Figures and Tables

**Table 1:** Demographics by group

Demographic	GAD	Anxiety control	Community
Total *n*	46	18	38
Age			
Mean	15.20	15.33	14.93
SD	1.43	1.64	2.29
Range	12.01-17.70	12.53-17.37	12.02-17.92
Gender, *n* (%)			
Male	2 (4.35)	10 (55.56)	21 (55.26)
Female	44 (95.65)	8 (44.44)	17 (44.74)
Non-binary	0 (0.00)	0 (0.00)	0 (0.00)
Socioeconomic status, *n* (%)			
Professional	23 (50.00)	9 (50.00)	23 (60.54)
Other employed	18 (39.13)	7 (38.89)	11 (28.95)
Unemployed, student, retired	3 (6.52)	1 (5.56)	2 (5.26)
Missing	0 (0.00)	1 (5.56)	0 (0.00)
Ethnicity, *n* (%)			
White British	41 (89.13)	16 (88.89)	0 (0.00)
Black and minority ethnic	5 (10.87)	2 (11.11)	0 (0.00)
Missing	0 (0.00)	0 (0.00)	38 (100.00)
Presence of diagnosis, *n* (%)			
GAD	46 (100.00)	0 (0.00)	-
Social Anxiety Disorder	39 (84.78)	8 (44.44)	-
Specific Phobia	7 (15.22)	5 (27.78)	-
Panic Disorder without Agoraphobia	1 (2.17)	0 (0.00)	-
Panic Disorder with Agoraphobia	2 (4.35)	1 (5.56)	-
Agoraphobia without Panic Disorder	9 (19.57)	2 (11.11)	-
Selective mutism	0 (0.00)	0 (0.00)	-
ADNOS	0 (0.00)	1 (5.56)	-
Separation Anxiety Disorder	8 (17.39)	1 (5.56)	-
Illness Anxiety Disorder	1 (2.17)	0 (0.00)	-
OCD	1 (2.17)	0 (0.00)	-
PTSD	1 (2.17)	0 (0.00)	-
Dysthymia	3 (6.52)	0 (0.00)	-
MDD	5 (10.87)	1 (5.56)	-
ADHD	2 (4.35)	1 (5.56)	-
Conduct Disorder	0 (0.00)	0 (0.00)	-
Oppositional Defiant Disorder	0 (0.00)	0 (0.00)	-
Number of diagnoses, mean (SD)	2.61 (1.27)	1.39 (0.50)	-
Primary anxiety disorder, *n* (%)			
GAD	18 (39.13)	0 (0.00)	-
Social Anxiety Disorder	24 (52.17)	8 (44.44)	-
Specific Phobia	1 (2.17)	5 (27.78)	-
Panic Disorder without Agoraphobia	0 (0.00)	0 (0.00)	-
Panic Disorder with Agoraphobia	0 (0.00)	1 (5.56)	-
Agoraphobia without Panic Disorder	1 (2.17)	2 (11.11)	-
Selective mutism	0 (0.00)	0 (0.00)	-
ADNOS	0 (0.00)	1 (5.56)	-
Separation Anxiety Disorder	1 (2.17)	1 (5.56)	-
Illness Anxiety Disorder	1 (2.17)	0 (0.00)	-
CSR of primary diagnosis			
Mean (SD)	5.93 (0.80)	6.18 (0.88)	-

**Note**. SD = Standard Deviation; GAD = Generalised Anxiety Disorder; ADNOS = Anxiety Disorder Not Otherwise Specified; OCD = Obsessive Compulsive Disorder; PTSD = Post-Traumatic Stress Disorder; MDD = Major Depressive Disorder; ADHD = Attention Deficit Hyperactivity Disorder. SES was based on parent/guardian occupation, which was coded accorded to the ONS Standard Occupational Classification [78]. Codes were subsequently collapsed into the categories displayed in Table 1. Where two parent/guardian occupations were provided, the highest category was used as an indication of the overall SES of the adolescent

**Table 2:** Descriptive statistics for symptom and cognitive aspect measures

Symptom or cognitive aspect measure		GAD	Anxiety control	Community control	Total
IUSC	*n*	46	18	38	102
	Mean (SD)	89.43 (22.96)	58.42 (20.88)	52.09 (20.15)	70.05 (27.81)
	Observed min - max	45-123	27-101	27-87	27-123
WW2	*n*	46	18	38	102
	Mean (SD)	55.49 (19.52)	38.26 (9.37)	43.06 (15.37)	47.82 (17.97)
	Observed min - max	26-98	25-58	25-87	25-98
CAQ	*n*	45	17	37	99
	Mean (SD)	76.81 (19.80)	62.59 (16.62)	52.16 (18.80)	65.15 (21.87)
	Observed min - max	27-111	37-94	25-89	25-111
RCADS-GAD	*n*	46	18	38	102
	Mean (SD)	10.48 (3.33)	6.78 (3.35)	4.92 (2.52)	7.75 (3.97)
	Observed min - max	5-18	1-11	1-10	1-18
RCADS-Depression	*n*	46	18	38	102
	Mean (SD)	15.24 (11.59)	11.50 (4.50)	7.21 (5.15)	11.59 (6.21)
	Observed min - max	3-28	4-19	1-22	1-28

**Note**. IUSC = Intolerance of Uncertainty Scale for Children; WW2 = Why Worry-II; CAQ = Cognitive Avoidance Questionnaire; RCADS-GAD = Revised Children Anxiety and Depression Scale - Generalised Anxiety Disorder subscale; RCADS-Depression = Revised Children Anxiety and Depression Scale - Depression subscale

**Table 3:** Linear regression results (GAD compared to anxiety control group)

		Dependent variable		
		IUSC	CAQ	WW2
Gender	Estimate (SE)	7.09 (7.89)	7.21 (7.39)	11.52 (6.17)
	*p*-value	0.371	0.333	0.067
Age	Estimate (SE)	2.50 (1.68)	-0.21 (1.57)	0.42 (1.32)
	*p*-value	0.142	0.895	0.751
Depression	Estimate (SE)	2.13 (0.50)	1.32 (0.46)	1.56 (0.39)
	*p*-value	< 0.001	0.005	< 0.001
Group	Estimate (SE)	-26.98 (7.01)	-13.06 (6.72)	-17.35 (5.50)
	*p*-value	<0.001	0.057^a^	0.003
Constant	Estimate (SE)	18.57 (26.95)	59.51 (25.12)	24.83 (21.13)
	*p*-value	0.494	0.021	0.245
	*n*	64	62	64
	R2	0.471	0.227	0.37
	Adjusted R2	0.435	0.172	0.328
	Residual SE	19.76 (df = 59)	18.11 (df = 57)	15.49 (df = 59)
	F Statistic	13.15 (df = 5, 59)	4.18 (df = 4, 57)	8.67 (df = 4, 59)

**Note**. SE = standard error; IUSC = Intolerance of Uncertainty Scale for Children; CAQ = Cognitive Avoidance Questionnaire; WW2 = Why Worry-II ^a^ Although this p-value is less than its Benjamini-Hochberg critical value, it is considered to be non-significant as it does not meet the pre-registered criteria of p < 0.05

**Table 4:** Linear regression results (GAD compared to community control group)

		Dependent variable		
		IUSC	CAQ	WW2^a^
Gender	Estimate			
	(SE)	-2.32 (5.33)	3.18 (5.15)	3.23 (4.58)
	*p*-value	0.014	0.538	0.587
Age	Estimate			
	(SE)	0.63 (1.06)	-0.59 (1.03)	0.11 (0.91)
	*p*-value	0.554	0.566	0.802
Depression	Estimate			
	(SE)	2.44 (0.38)	1.82 (0.37)	1.80 (0.33)
	*p*-value	< 0.001	< 0.001	< 0.001
Group	Estimate			
	(SE)	-16.43 (5.64)	-11.77 (5.41)	0.44 (4.84)
	*p*-value	0.005	0.033	0.929
Constant	Estimate			
	(SE)	42.82 (16.98)	57.88 (16.39)	26.18 (14.57)
	*p*-value	0.014	< 0.001	< 0.001
	*n*	84	82	84
	R2	0.627	0.466	0.362
	Adjusted R2	0.608	0.439	0.329
	Residual SE	17.88 (df = 79)	17.12 (df = 77)	15.34 (df = 79)
	F Statistic	33.22 (df = 4, 79)	16.82 (df = 4, 77)	11.19 (df = 4, 79)

**Note**. SE = standard error; IUSC = Intolerance of Uncertainty Scale for Children; CAQ = Cognitive Avoidance Questionnaire; WW2 = Why Worry-II

a Due to heteroscedasticity in raw scores, a log transform was successfully applied to WW2 scores in order to satisfy the assumptions of the statistical test

**Table 5:** Bootstrapped multiple linear regression results with GAD symptoms as the outcome variable

	Beta co-efficient estimate	95% CI	*p*-value
Depression	0.19	-0.06-0.44	0.143
CAQ	0.01	-0.04-0.07	0.702
IUSC	-0.02	-0.08-0.05	0.645
WW2	0.03	-0.04-0.11	0.407

**Note**. CI = confidence interval; CAQ = Cognitive Avoidance Questionnaire score, IUSC = Intolerance of Uncertainty - Child total score; WW2 = Why Worry-II score

## Data Availability

Due to the sensitive nature of the questions asked in this survey, participants were assured that raw data would remain confidential. Therefore, data are not publicly available.

## Summary

This study provides preliminary evidence for the broad specificity of intolerance of uncertainty to GAD in a clinical sample of adolescents. Youth with primary or secondary GAD were significantly more intolerant of uncertainty than youth with other anxiety disorders, and non-anxious community participants. Meanwhile, cognitive avoidance was similarly elevated in both clinical groups relative to community participants. The GAD group reported significantly higher positive beliefs about worry compared to the anxiety control group, but the difference between the GAD and community groups on this measure was non-significant. Our study offers a theoretical basis for further research into the cognitive mechanisms driving GAD in adolescents, with particular support for the relevance of intolerance of uncertainty.

## Footnotes

1 The pre-registration document listed ANCOVA as the analysis approach for Hypotheses 1 and 2. However, given the unsuitability of the data for this statistical method, regression analyses were conducted instead. No other changes from the pre-registered plan were made.
