# Supplementary material for: Cognitive Aspects of Generalised Anxiety Disorder in Adolescents: Exploring Intolerance of Uncertainty, Cognitive Avoidance, and Positive Beliefs About Worry
Source: Child Psychiatry Hum Dev. 2025 Feb 25;57(5):1518–31. doi: 10.1007/s10578-025-01809-3 (PMC7618352; doi:10.1007/s10578-025-01809-3)
Supplement: Supplementary file 1 [file 10578_2025_1809_MOESM1_ESM.docx]

## **Cognitive Aspects of Generalised Anxiety Disorder in Adolescents: Exploring Intolerance of Uncertainty, Cognitive Avoidance, and Positive Beliefs About Worry**

## **Supplementary Materials**

**Table S1**

*Missing data*

|  | Number of participants who failed to answer one or more item | | |
| --- | --- | --- | --- |
|  | **GAD (n=46)** | **Anxiety control (n=18)** | **Community (n=38)** |
| IUSC | 1 | 1 | 4 |
| CAQ | 9 | 1 | 1 |
| WW2 | 3 | 4 | 1 |
| RCADS | 0 | 0 | 0 |
| Note. IUSC = Intolerance of Uncertainty Scale for Children; CAQ = Cognitive Avoidance Questionnaire; WW2 = Why Worry-II; RCADS = Revised Children's Anxiety and Depression Scale | | | |

For the IUSC, 6 participants (1 in the GAD group, 1 in the anxiety control group, and 4 in the community group) failed to answer one item (3.70% of the scale). No participants missed more than 1 item. For the WW2, 7 participants failed to answer 1 item (3 in the GAD group, 3 in the anxiety control group, and 1 in the community group), constituting 4.00% of the scale, and 1 participant in the anxiety control group had 2 items missing (8.00% of the scale). For the CAQ, 8 participants in the GAD group failed to answer 1 item (4.00% of the scale). Three participants (1 from each group) had more than 10% missing data, and were excluded from analyses.

**Table S2**

*Benjamini-Hochberg method applied to results of primary and exploratory hypotheses*

| Primary hypotheses |  | |  |  |  |
| --- | --- | --- | --- | --- | --- |
| **Hypothesis** | **Dependent variable** | | ***p***-value | **Rank** | **(i/m)Q** |
| 1 | IUSC | | 0.000296* | 1 | 0.0167 |
| 1 | WW2 | | 0.00252* | 2 | 0.0333 |
| 2 | IUSC | | 0.00464* | 3 | 0.0500 |
| 2 | CAQ | | 0.0327* | 4 | 0.0667 |
| 1 | CAQ | | 0.0567^a^ | 5 | 0.0833 |
| 2 | WW2 | | 0.929 | 6 | 0.1000 |
|  |  | |  |  |  |
| Exploratory hypotheses | |  |  |  |  |
| **Hypothesis** | **Dependent variable** | | ***p***-value | **Rank** | **(i/m)Q** |
| 1 | IUSC | | 0.02135 | 1 | 0.0167 |
| 1 | WW2 | | 0.09016 | 2 | 0.0333 |
| 2 | CAQ | | 0.1084 | 3 | 0.0500 |
| 1 | CAQ | | 0.184 | 4 | 0.0667 |
| 2 | IUSC | | 0.184 | 5 | 0.0833 |
| 2 | WW2 | | 0.35052 | 6 | 0.1000 |
|  |  | |  |  |  |
| ***Note.*** *i = the individual p-value's rank; m = total number of tests; Q = false discovery rate (10%).*  ** indicates significance according to Benjamini-Hochberg criteria (i.e., largest p-value for which p < (i /m)Q is significant, and all ranked p-values smaller than the largest value are also significant).*  *^a^Although this p-value is smaller than its critical value, it is interpreted as non-significant as it does not meet the pre-registered criteria for significance (p < 0.05).* | | | | | |

**Table S3**

*Exploratory linear regression results (primary GAD group (n = 18) compared to anxiety control group)*

|  |  |  | Dependent variable |  |
| --- | --- | --- | --- | --- |
|  |  | IUSC | CAQ | WW2^a^ |
| Gender | Estimate (SE) | 8.21 (10.19) | 0.85 (9.28) | -0.003 (0.004) |
|  | *p*-value | 0.426 | 0.928 | 0.440 |
| Age | Estimate (SE) | 3.33 (2.20) | 2.21 (1.98) | -0.001 (0.001) |
|  | *p*-value | 0.141 | 0.273 | 0.439 |
| Depression | Estimate (SE) | 1.92 (0.69) | 1.17 (0.61) | -0.0003 (0.0002) |
|  | *p*-value | 0.010** | 0.067 | 0.301 |
| Group | Estimate (SE) | -22.11 (9.12) | -11.38 (8.67) | 0.006 (0.003) |
|  | *p*-value | 0.021^b^ | 0.184 | 0.090 |
| Constant | Estimate (SE) | 2.97 (34.61) | 26.55 (31.04) | 0.036 (0.012) |
|  | *p*-value | 0.932 | 0.399 | 0.006** |
|  | *n* | 26 | 35 | 36 |
|  | R2 | 0.373 | 0.234 | 0.142 |
|  | Adjusted R2 | 0.292 | 0.132 | 0.031 |
|  | Residual SE | 20.94 (df = 31) | 18.51 (df = 30) | 0.007 (df = 31) |
|  | F Statistic | 4.61 (df = 4, 31) | 2.30 (4, 30) | 1.28 (df = 4, 31) |
| ***Note.*** *SE = standard error; IUSC = Intolerance of Uncertainty Scale for Children; CAQ = Cognitive Avoidance Questionnaire; WW2 = Why Worry-II. *p<0.05, **p<0.01, ***p<0.001* | | | | |
| *^a^ Due to heteroscedasticity in raw scores, an inverse transform was successfully applied to WW2 scores in order to satisfy the assumptions of the statistical test.* | | | | |
| *^b^ This result did not retain significance after applying the Benjamini-Hochberg method.* | | | | |

**Table S4**

*Exploratory linear regression results (primary GAD group (n = 18) compared to community control group)*

|  |  |  | Dependent variable |  |
| --- | --- | --- | --- | --- |
|  |  | IUSC | CAQ | WW2a |
| Gender | Estimate (SE) | -3.88 (5.80) | -0.85 (5.62) | -0.02 (0.10) |
|  | *p*-value | 0.506 | 0.880 | 0.860 |
| Age | Estimate (SE) | 0.54 (1.15) | 0.27 (1.11) | 0.01 (0.02) |
|  | *p*-value | 0.642 | 0.808 | 0.74 |
| Depression | Estimate (SE) | 2.41 (0.45) | 1.91 (0.43) | 0.03 (0.01) |
|  | *p*-value | < 0.001*** | < 0.001*** | <0.001*** |
| Group | Estimate (SE) | -8.86 (6.58) | -10.33 (6.32) | 0.11 (0.22) |
|  | *p*-value | 0.184 | 0.108 | 0.351 |
| Constant | Estimate (SE) | 37.75 (18.08) | 45.08 (17.49) | 3.29 (0.31) |
|  | *p*-value | 0.042* | 0.013 | < 0.001*** |
|  | *n* | 56 | 55 | 56 |
|  | R2 | 0.535 | 0.464 | 0.271 |
|  | Adjusted R2 | 0.498 | 0.421 | 0.214 |
|  | Residual SE | 17.69 (df = 51) | 16.98 (df = 50) | 0.30 (df = 51) |
|  | F Statistic | 14.64 (df = 4, 51) | 10.80 (df = 4, 50) | 4.74 (df = 4, 51) |
| ***Note.*** *SE = standard error; IUSC = Intolerance of Uncertainty Scale for Children; CAQ = Cognitive Avoidance Questionnaire; WW2 = Why Worry-II. *p<0.05, **p<0.01, ***p<0.001* | | | | |
| *a Due to heteroscedasticity in raw scores, a log transform was successfully applied to WW2 scores in order to satisfy the assumptions of the statistical test.* | | | | |
